# Body Mass and White Matter Integrity: The Influence of Vascular and Inflammatory Markers

**DOI:** 10.1371/journal.pone.0077741

**Published:** 2013-10-16

**Authors:** Brianne Magouirk Bettcher, Christine M. Walsh, Christa Watson, Joshua W. Miller, Ralph Green, Nihar Patel, Bruce L. Miller, John Neuhaus, Kristine Yaffe, Joel H. Kramer

**Affiliations:** 1 University of California, San Francisco, Neurology Department, Memory and Aging Center, San Francisco, California, United States of America; 2 Stanford University, Department of Psychiatry, Neuropsychology and Neuroimaging Laboratory, Palo Alto, California, United States of America; 3 Rutgers University, Department of Nutritional Sciences, New Brunswick, New Jersey, United States of America; 4 University of California Davis Medical Center, Department of Pathology and Laboratory Medicine, Sacramento, California, United States of America; 5 University of California, San Francisco, Department of Psychiatry, Neurology, and Epidemiology and Biostatistics, San Francisco, California, United States of America; INRCA, Italy

## Abstract

High adiposity is deleteriously associated with brain health, and may disproportionately affect white matter integrity; however, limited information exists regarding the mechanisms underlying the association between body mass (BMI) and white matter integrity. The present study evaluated whether vascular and inflammatory markers influence the relationship between BMI and white matter in healthy aging. We conducted a cross-sectional evaluation of white matter integrity, BMI, and vascular/inflammatory factors in a cohort of 138 healthy older adults (mean age: 71.3 years). Participants underwent diffusion tensor imaging, provided blood samples, and participated in a health evaluation. Vascular risk factors and vascular/inflammatory blood markers were assessed. The primary outcome measure was fractional anisotropy (FA) of the genu, body, and splenium (corpus callosum); exploratory measures included additional white matter regions, based on significant associations with BMI. Regression analyses indicated that higher BMI was associated with lower FA in the corpus callosum, cingulate, and fornix (*p*<.001). Vascular and inflammatory factors influenced the association between BMI and FA. Specifically, BMI was independently associated with the genu [β=-.21; B=-.0024; 95% CI, -.0048 to -.0000; *p*=.05] and cingulate fibers [β=-.39; B=-.0035; 95% CI,-.0056 to -.0015; *p*<.001], even after controlling for vascular/inflammatory risk factors and blood markers. In contrast, BMI was no longer significantly associated with the fornix and middle/posterior regions of the corpus callosum after controlling for these markers. Results partially support a vascular/inflammatory hypothesis, but also suggest a more complex relationship between BMI and white matter characterized by potentially different neuroanatomic vulnerability.

## Introduction

Greater adiposity is associated with a myriad of negative consequences related to brain health [1–6], and serves as an important risk factor for the development of Alzheimer’s disease [7–9]. Level of adiposity has increasingly been implicated in structural brain changes [3,4] and longitudinal atrophy rates [5,6] associated with both aging and neurodegenerative disease; however, the mechanisms of action and regional specificity of these neuroanatomical relationships remain poorly understood. In particular, a dearth of research exists on the association between body mass (BMI) and white matter structure in the brain, despite the well-documented classification of obesity as a vascular risk factor [10,11]. Recent literature suggests that BMI associates with global decreases in white matter integrity [12] and alterations in corpus callosum diffusivity markers [13,14]. While these studies point to adiposity-associated myelin or axonal degeneration, it is unclear whether this relationship is independent of traditional vascular risk factors. Furthermore, given the strong association between BMI and both inflammatory/vascular mediators [15,16], a focused examination of possible mechanisms for white matter alterations is needed.

In the current study, we examined the association between BMI, vascular/inflammatory indices, and white matter integrity using diffusion tensor imaging. Considering that alterations in the corpus callosum have been linked to BMI [17] and broadly to vascular risk factors [18,19], we chose this region as a primary ROI; however, to inclusively assess the possibility of more extensive alterations, we also examined associations with white matter regions throughout the brain. To provide an examination of possible mechanisms that may influence the relationship between BMI and white matter integrity, we examined the stepwise impact of controlling for vascular risk factors and blood markers of inflammation and vascular health on this association.

## Methods

### Participants

A sample of 138 neurologically healthy, community dwelling older adult participants was selected from the University of California, San Francisco Memory and Aging Center database based on the availability of stored blood specimens and diffusion tensor imaging. Both the blood draw and neuroimaging session occurred within a 60-day period. Participants (ages: 60-95) were reviewed in a screening visit, which entailed an informant interview, neurological examination, and cognitive testing. Inclusion was based on several criteria, including a Mini-Mental State Exam score ≥26, Clinical Dementia Rating score of 0, and no subject or informant report of cognitive decline during the previous year. Participants were excluded if they had a major psychiatric disorder, neurological condition affecting cognition (e.g. large vessel infarct), dementia or mild cognitive impairment, substance abuse, systemic medical illnesses (e.g. cancer), or current depression (Geriatric Depression Scale Score≥15) [20]. Demographic and history variables are reported in Table 1. In terms of ethnic and racial demographics, our sample was 88.4% white, 3.6% Asian, and 1.4% Hispanic. Approximately 6.5% of our sample did not report their race or ethnicity. The study was approved by the UCSF committee on human research, and all participants provided written, IRB-approved informed consent before participating. In compliance with data sharing plans, data files are available upon request to the corresponding author. 

### Measures

#### Body mass

Body mass index (BMI) was calculated as follows: [weight (kg)/height (m)^2^]. BMI was analyzed as a continuous variable. Notably, 45% of our participants were in the healthy weight range (BMI between 18.5-24.90), 43% were overweight (BMI between 25.00 and 29.90), 1% was underweight (BMI less than 18.5), and 11% were categorized as obese (BMI ≥30.00) according to standards established by the Center for Disease Control. 

#### Vascular risk factors

All available information regarding medical and health history was garnered via participant self-report. Health variables included history (yes/no) of the following: smoking, hypertension, hypercholesterolemia, and diabetes. 

#### Diffusion tensor imaging

MRI scans were obtained on a 3.0 Tesla Siemens (Siemens, Iselin, NJ) TIM Trio scanner equipped with a 12-channel head coil located at the UCSF Neuroscience Imaging Center. Whole brain images were acquired using volumetric magnetization prepared rapid gradient-echo sequence (MPRAGE; TR/TE/TI = 2300/2.98/900 ms, α = 9°). Diffusion imaging data were acquired via a spin-echo, echo planar imaging sequence with 55 slices 2 mm thick (TR/TE = 8000/109 ms, FOV= 220 mm, matrix = 100 x 100) in two series. 

DTI data were preprocessed and analyzed using FMRIB’s Diffusion Toolbox (FDT) and tract based spatial statistics (TBSS) from the fMRI Software Library (FSL 4.1.6; http://fsl.fmrib.ox.ac.uk/fsl) [21]. First, raw data were corrected for head movement and eddy current distortions using FDT. Brain extraction and binary brain mask creation took place using the b0 image through the FSL Brain Extraction Tool (BET). Fractional Anisotropy (FA) maps were created based on the diffusion tensor modeling results from FSL DTIFIT. All subjects' FA data were registered using the nonlinear registration tool FNIRT [22] to the IXI Aging DTI Template [23] masked by a study-specific averaged image. The mean FA and mean FA skeleton were created from the study sample. Each subject's aligned FA data was then projected onto the mean FA skeleton. For the purposes of the current study, we chose to focus solely on FA values, as they are the most commonly reported diffusion metric and provide a summary measure of white matter fiber integrity.

We employed the JHU ICBM-DTI-81 white matter labels [24] to label and mask areas of the white matter skeleton. Mean FA values for each white matter region was calculated using the FSL utility fslstats. We selectively examined the genu, body, and splenium of the corpus callosum as our primary outcome measures. This decision was based on recent literature linking vascular factors to integrity of both the whole and subregions of the corpus callosum [14,19,25]. In addition, BMI has also recently been linked to alterations in the corpus callosum [17]. For the purposes of inclusivity, we also examined additional white matter regions associated with cognitive aging, including: anterior, superior and posterior corona radiata; posterior thalamic radiation; anterior and posterior limb of the internal capsule; cerebral peduncles; superior longitudinal fasciculus; superior fronto-occipital *Fasciculus*; uncinate fasciculus; sagittal stratum; external capsule; cingulate and hippocampal sections of the cingulum; and the fornix (body and stria terminalis). All ROI’s represent a mean of both left and right hemisphere structures.

#### Laboratory measures

Blood was collected into serum separator tubes and left to clot at room temperature for 30–60 min, and into EDTA plasma tubes. The blood was then centrifuged at 2500 rpm at room temperature for 15 min. Plasma and serum were stored at −80 °C until analysis. The vascular and inflammatory peripheral measures were selected based on a rich literature linking them with obesity [1,10,15,16,26]. Specifically, interleukin-6 (IL-6) was measured using a Quantikine ELISA kit from R&D systems (Minneapolis, MN). Homocysteine was measured by HPLC with post-column fluorescence detection [27]. Il-6 and homocysteine were measured in the laboratory of Dr. Ralph Green at the University of California Davis Medical Center (Sacramento, CA). The following clinical laboratory measures were assessed in the University of California Davis Medical Center Clinical Laboratory: high-density lipoprotein (HDL), low-density lipoprotein (LDL), high sensitivity C-reactive protein (hsCRP), creatinine, glucose and insulin. The homeostasis model of assessment-insulin resistance (HOMA-IR) ratio was calculated [(fasting insulin (µU/mL)× fasting glucose (mg/dL))/405] and used in lieu of individual insulin and glucose levels. 

Of note, one participant presented with elevated hsCRP levels (i.e. 24.1 mg/L); as such, his data was removed from further analyses due to concerns that the individual may have had an acute inflammatory or an underlying chronic illness [28]. In addition, 3 individuals did not have enough blood sample to run all laboratory measures; as such, the sample size was lower (n=135) for analyses controlling for blood markers. 

### Statistical Analyses

Associations between blood markers, demographics, and white matter ROI’s were calculated using Pearson partial correlations, controlling for age. First, the genu, body, and splenium FA were correlated with BMI; we then calculated additional correlations with the remaining 15 white matter regions FA values. These regions were included as outcome variables of interest if their r_p_ values were greater than .20 (a small to medium effect size).

Demographic and blood marker variables were identified as covariates for regression models if they were significantly associated with either the predictor variable (BMI) or primary ROI (CC). Using these criteria, lower integrity of the CC was associated with: higher age; a history of hypertension; higher levels of IL-6 and creatinine; and a higher HOMA-IR ratio. Similarly, larger BMI was associated with male gender; history of ever smoking, hypertension and hypercholesterolemia; higher levels of hsCRP, IL-6, and HDL; and a higher HOMA-IR ratio. Education, race, homocysteine, and LDL were not related to BMI or CC integrity in our sample. In addition, a history of diabetes was not associated with BMI or CC integrity; however, only four individuals in our sample had been diagnosed with diabetes, thereby yielding a limited range. As such, these five variables were not included as covariates in the regression analyses.

#### Multiple regressions, primary analyses

Using regression modeling, we examined four models of prediction for each of our corpus callosum ROI’s, with BMI serving as the predictor of interest. In the first model, we included BMI as the sole predictor, with our white matter ROI’s as the outcome measures. In the second model, we added age and gender as covariates. In the third model, we added history of hypertension, ever smoking, and hypercholesterolemia as covariates. Finally, the fourth model controlled for hsCRP, IL-6, HDL, creatinine, and HOMA-IR as well as the aforementioned covariates. Considering that FA values are less than 1.00, confidence intervals (CI’s) are reported with four decimal points.

#### Multiple regressions, exploratory analyses

The aforementioned models were also conducted with the remaining white matter ROI’s found to be significantly related to BMI. 

All statistical analyses were conducted using SAS 9.3.

## Results

### BMI and White Matter Correlations (Figure 1)

**Figure 1:**
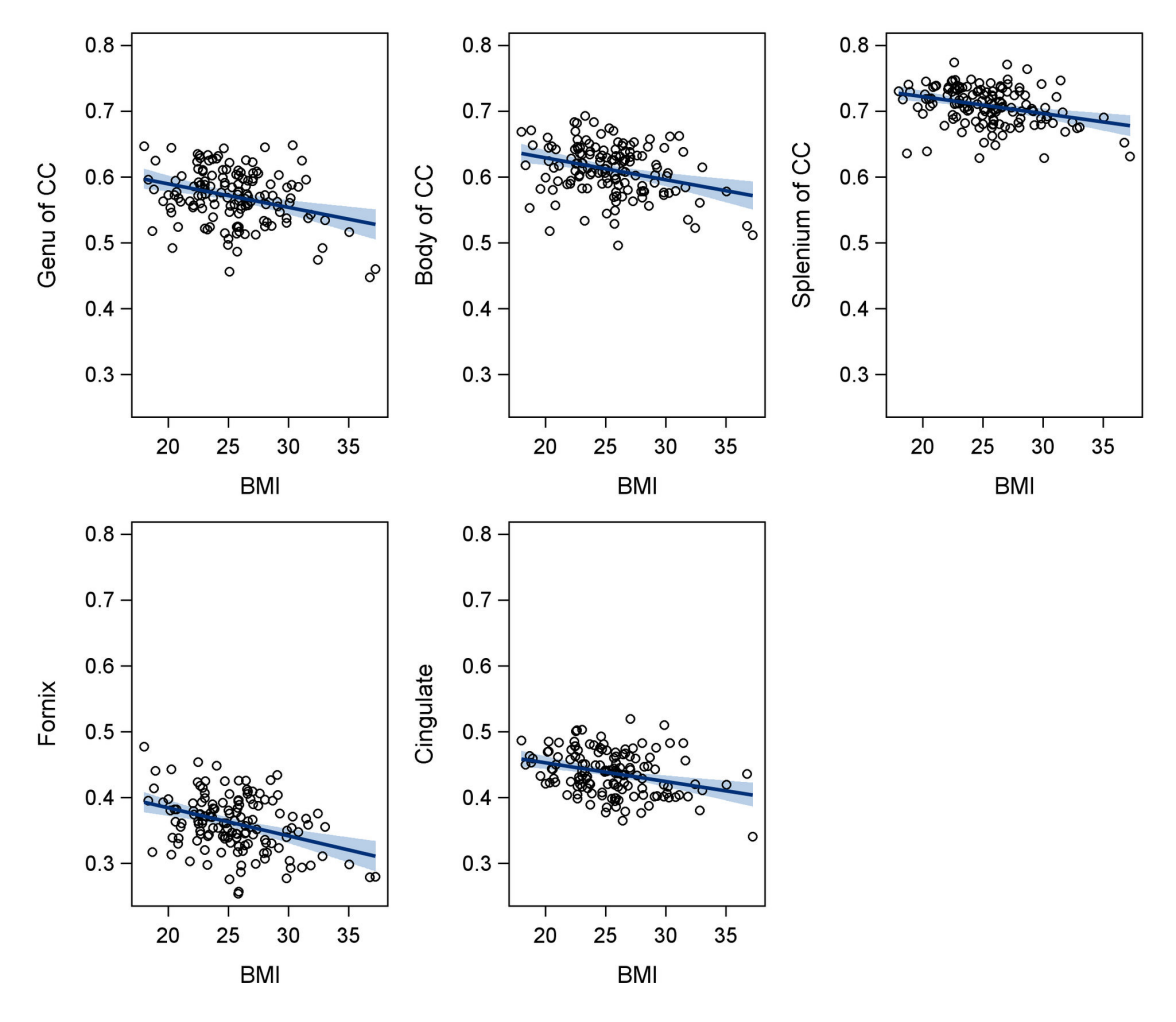
Displays the unadjusted relationship between BMI and fractional anisotropy (FA) of the corpus callosum genu, body, and splenium (top row), and FA of the cingulum and fornix (bottom row), with regression lines. Shaded areas represent 95% confidence intervals. All correlations displayed are significant at *p*<.05.

 When controlling for age, BMI was significantly related to the genu (*r*_p_=-.30, *p*<.001), body (*r*_p_=-.30, *p*<.001), and splenium (*r*_p_=-.31, *p*<.001) of the corpus callosum. Partial correlations with the remaining 15 white matter areas yielded only two additional areas with an *r*_p_ greater than .20: the cingulate section of the cingulum (*r*_p_=-.30, *p*<.001), and the fornix (*r*_p_=-.35, *p*<.001). Figure 1 displays the unadjusted associations between BMI and these regions. Partial correlations between BMI and the remaining white matter regions yielded *r*_p_’s less than .20 (see Table S1). 

### Primary Analyses: BMI and CC, Multiple Regressions (Table 2)

#### Model 1: BMI and CC integrity (FA)

As shown in Table 2, BMI was inversely associated with the genu (t=-3.85), body (t=-3.81), and splenium (t=-3.96) FA of the corpus callosum in the unadjusted model. 

#### Model 2: BMI and CC integrity (FA), controlling for demographic variables

After controlling for demographic variables (age and gender), the association between BMI and the genu (t=-3.32), body (t=-3.22), and splenium (t=-3.31) of the corpus callosum remained relatively robust. 

#### Model 3: BMI and CC integrity (FA), controlling for demographic variables and vascular risk factors

After controlling for demographic variables and vascular risk factors (history of: hypertension, ever smoking, hypercholesterolemia) all associations between BMI and the genu (t=-3.36), body (t=-2.41), and splenium (t=-3.11) remained significant.

#### Model 4: BMI and CC integrity (FA), controlling for demographic variables, vascular risk factors, and inflammatory and vascular blood markers

After controlling for demographic variables, vascular risk factors, and inflammatory and vascular blood markers (hsCRP, IL-6, HDL, creatinine, HOMA-IR), differential patterns of associations emerged. The association between BMI and the genu remained significant in the model (t=-1.98); however, BMI was no longer a significant predictor of the body (t=-1.59) or the splenium (t=-1.86; trend) in the model. 

### Exploratory Analyses: BMI, Cingulate, and the Fornix (see Table 2)

 The same regression models were conducted using the cingulate and the fornix as separate outcome variables, based on the correlation analyses previously described. BMI was significantly related to the cingulate (t=-3.79) and fornix (t=-4.51) in Model 1. Upon controlling for demographic variables in Model 2, both relationships remained significant (Cingulate, t=-4.04; Fornix, t=-3.73). After controlling for demographic variables and vascular risk factors in Model 3, BMI continued to significantly relate to the cingulate (t=-3.71) and the fornix (t=-2.97). Finally after controlling for all variables in Model 4, BMI remained significantly associated with the cingulate (t=-3.42), with the fornix demonstrating a trend (t=-1.87). 

## Discussion

 The present study examined the relationship between BMI and white matter integrity, while controlling for putative risk factors and vascular and inflammatory blood markers. Our findings suggest that higher BMI is differentially related to lower integrity, as indexed by fractional anisotropy, of our primary regions of interest: the genu, body, and splenium of the corpus callosum. The associations between BMI and the corpus callosum are consistent with recent DTI studies focused on young adults [29] and lifespan approaches [13]. Follow-up exploratory analyses also yielded significant negative associations between BMI and the cingulate, as well as BMI and the fornix. Upon conducting a focused examination of possible confounders of the BMI-white matter correlation, disparate relationships emerged. Specifically, the association between BMI and the body (CC), splenium (CC), and fornix was influenced by vascular and inflammatory factors, such that controlling for risk factors and blood markers eradicated the significant association between BMI and these ROI’s. In contrast, BMI remained associated with the genu (CC) and cingulate, with higher BMI contributing to lower white matter integrity in these ROI’s over and above demographic variables and vascular/inflammatory factors. To our knowledge, this study provides the first evaluation of both risk factors and blood markers that may influence the relationship between BMI and white matter in healthy older adults. The results highlight important implications for public health, as while larger body mass has frequently been associated with cardiovascular sequelae, our study suggests a striking link between BMI and microstructural brain health. 

 While several studies underscore an association between BMI and brain structure, the majority of research has focused on grey matter correlates [5,6], and has not addressed the potentially influential role of vascular/inflammatory factors in subserving this relationship. Our findings suggest that the different vascular/inflammatory patterns underlying the relationship between BMI and white matter integrity may reflect regional susceptibility to metabolic dysfunction. In particular, posterior regions of the CC and the fornix may be particularly vulnerable, as the relationship between these regions and BMI were diminished upon controlling for vascular and immune factors. Considering that the hippocampus and fornix have been shown to be markedly sensitive to metabolic demands [30,31] and inflammatory processes [32,33], it is not surprising that vascular and inflammatory factors primarily accounted in part for the correlation between BMI and the fornix in our study. 

 An unexpected finding from the present study is that the associations between BMI and both the genu of the CC and the cingulate fibers were independent of risk factors and vascular/inflammatory blood markers. This finding was not noted for the body or splenium of the CC. Previous studies have suggested that vascular risk factors are associated with the entirety of the CC [17,19], whereas degenerative factors (i.e. AD diagnosis) are disproportionately associated with the posterior CC [19]. Stemming from this conclusion, one hypothesis based on our findings is that the metabolic relationship between BMI and white matter is buttressed by an underlying Alzheimer’s disease (AD) process that has not yet manifested clinically; in other words, the strong role of vascular/inflammatory factors in bolstering the association between BMI and specific white matter regions may serve as a proxy for an early degenerative disease. A predilection for white matter regions associated with AD is only partially supported by our data. In support of this explanation, the relationship between BMI and both the fornix and posterior regions of the corpus callosum were influenced by vascular and inflammatory factors in the present study. Conversely, an additional region associated with AD, the cingulate [34], remained significantly related to BMI after controlling for vascular blood markers, suggesting that additional markers not measured in the current study may be driving this association.

 A more parsimonious explanation for our findings is that the corpus callosum, fornix, and cingulate are all sensitive regions to the downstream vascular and inflammatory cascade associated with greater adiposity. This is particularly relevant to address given the overlapping confidence intervals for the BMI-white matter associations, and the noted trends between BMI and both the splenium and fornix. Additional measures of adiposity and vascular stress may further mediate these relationships, particularly in anterior white matter regions that are known to be vulnerable to the aging process [35,36]. Specifically, several indices of endothelial dysfunction (cell adhesion molecules), oxidative stress (ROS), and adipokines (adiponectin; leptin) were not assessed in the current study, and may contribute the aforementioned relationships between BMI and white matter integrity. In particular, recent studies with rats suggest that chronic hypoperfusion to the brain is related to glial changes and endothelial dysfunction in cerebral small vessels, particularly in frontal white matter regions [37,38]. As such, comprehensive assessment of factors that index endothelial functioning of the white matter may offer further understanding of the differential mechanisms by which BMI impacts white matter integrity in the brain.

 Additional caution is warranted in interpreting the role of vascular vs. inflammatory factors in the present study. Given the overlapping and often controversial role of vascular and inflammatory markers, we chose not to focus on the relative contribution of each. Specifically, several of our blood markers could be interpreted as both inflammatory and vascular in nature (e.g. hsCRP) [39,40]. In addition, identifying which vascular risk factors or blood markers primarily influenced the relationship between BMI and white matter integrity was not addressed in the current work for several reasons. First, our primary goal was to determine whether BMI independently associated with white matter integrity, even after accounting for traditional risk factors and blood markers. Thus, vascular and inflammatory factors were treated as confounds rather than primary variables of interest. Second, the blood markers used in the study involve related pathways and are correlated with each other; thus, parsing out the individual contribution of each in a stepwise manner may by obscured by their interrelatedness. 

 A limitation of the present study includes the relatively low rate of obesity in our sample. Although our data clearly suggests a linear association between BMI and white matter integrity (Figure 1), the modulatory role of vascular and inflammatory blood markers may differ in individuals who are obese. Despite this limitation, it is notable that our findings are robust in a relatively healthy cohort, and may even underestimate the relationship between adiposity and white matter integrity in overweight and obese older adults. In addition, we used BMI as our primary index of adiposity, which may not fully assess the role of adipose tissue in conferring risk for structural brain alterations; as such, additional measures such as hip-to-waist ratio, waist circumference, and body composition (e.g. Dual-energy X-ray absorptiometry) may be beneficial in future studies of adiposity and white matter changes. It’s also important to highlight that vascular risk factors were assessed using self-report measures, and thus were not based on study measurement. Although our inclusion of additional laboratory values of vascular health represents a strength of the study, inclusion of self-report indices may introduce some bias into the sample. 

In terms of the generalizability of our study results, we employed correlation analyses to identify potential covariates for subsequent regressions; although this approach reduced the number of regression analyses we conducted, it is unclear how this might impact replication of results using different samples. Thus, inclusion of different covariates in another study might alter the overall findings. We also did not conduct a formal multiple comparison adjustment, given that it would alter the statistical significance of only one comparison. Specifically, if we employed a Bonferroni family wise error correction (FWE), our genu-BMI findings would not have reached statistical significance in the final model. While this may underscore less regional variability to downstream vascular and inflammatory processes, considering the limited research on this topic we elected to report all findings without a conservative FWE correction.

 In summary, the present study offers a systematic evaluation of the relationship between BMI and white matter integrity in the brain using DTI, and provides a stepwise assessment of vascular and inflammatory factors that may contribute to this association in healthy older adults. Our findings suggest that vascular and inflammatory factors influenced the association between BMI and white matter fractional anisotropy. Specifically, BMI independently associated with the genu and cingulate fibers, even after controlling for traditional vascular risk factors and blood markers. In contrast, BMI was no longer associated with the fornix and middle/posterior regions of the corpus callosum after controlling for these markers. The complex and noxious connection between BMI and white matter integrity is strengthened in part by traditional vascular and inflammatory markers; however, regional variability also suggests the possibility of additional mechanisms driving this relationship. Extensive knowledge of these mechanisms will further clarify our understanding of cognitive aging and neurodegenerative diseases, and provide potential targets for therapeutic intervention.

**Table 1:** Demographic and Health Profiles.

	**Mean (SD)**	**Range**
Age (years)	71.3 (6.0)	(62-92)
Education (years)	17.6 (2.1)	(12-22)
Female Sex (%)	58.7%	
MMSE	29.3 (0.9)	(26-30)
Body Mass Index	25.4 (3.6)	(18.0-37.2)
History of Hypertension (%)	41.3%	
Ever Smoker (%)	50.7%	
History of Hypercholesterolemia (%)	52.9%	
hsCRP (mg/L)	1.6 (1.7)	(0.1-10.8)
IL-6 (pg/ml)	1.9 (2.2)	(0.0-18.0)
HOMA-IR Ratio	2.7 (2.2)	0.4-13.9
HDL (mg/dL)	64.6 (21.4)	31-130
LDL (mg/dL)	121.9 (41.8)	49-349
Creatinine (mg/dL)	0.9 (0.3)	0.5-3.1
Homocysteine (umol/L)	8.4 (3.5)	3.5-30.5

**Table 2:** BMI Regression Models for Corpus Callosum, Cingulate, and Fornix Fractional Anisotropy.

	Standardized β,*p*-value			
	Beta [95% Confidence Interval]			
	**Model 1:**	**Model 2:**	**Model 3:**	**Model 4:**
	**No Covariates**	**Demographics**	**Demographics, Vascular Risk**	**Demographics, Vascular Risk, Inflammatory/Vascular Markers**
**Genu**				
BMI	**β=-.31, *p*<.001**	**β=-.26, *p*=.001**	**β=-.28, *p*=.001**	**β=-.21, *p*=.05**
	**B=-.0036 [-.0055, -.0018]**	**B=-.0030 [-.0048, -.0012]**	**B=-.0032 [-.0051, -.0013]**	**B=-.0024 [-.0048, -.0000]**
**Body**				
BMI	**β=-.31, *p*<.001**	**β=-.25, *p*=.002**	**β=-.20, *p*=.02**	β=-.17, *p*=.12
	**B=-.0033 [-.0051, -.0016]**	**B=-.0027 [-.0044, -.0011]**	**B=-.0022 [-.0040, -.0004**]	B=-.0018 [-.0041, .0004]
**Splenium**				
BMI	**β=-.32, *p*<.001**	**β=-.26, *p*=.001**	**β=-.27, *p*=.002**	β=-.20, *p*=.07
	**B=-.0026 [-.0039, -.0013]**	**B=-.0021 [-.0034, -.0008**]	**B=-.0022 [-.0035, -.0008]**	B=-.0016 [-.0033, .0001]
**Cingulate**				
BMI	**β=-.31, *p*<.001**	**β=-.34, *p*<.001**	**β=-.34, *p*<.001**	**β=-.39, *p*<.001**
	**B=-.0028 [-.0043, -.0014]**	**B= -.0031 [-.0046, -.0012]**	**B=-.0031 [-.0047, -.0014]**	**B=-.0035 [-.0056, -.0015]**
**Fornix**				
BMI	**β=-.36, *p*<.001**	**β=-.28, p<.001**	**β=-.24; *p*=.004**	β=-.19; *p*=.06
	**B=-.0043 [-.0061, -.0024]**	**B=-.0033 [-.0051, -.0016]**	**B=-.0029 [-.0048, -.0010]**	B=-.0022 [-.0047, .0001]

Regression models, controlling for additional vascular and inflammatory factors at each step. Each model displays the relationship between Body Mass Index (BMI) and fractional anisotropy (FA) of white matter in the brain. Model 1 includes no covariates. Model 2 adjusts for demographic variables (i.e. age, gender). Model 3 adjusts for demographic variables, as well as vascular risk factors (i.e. history of hypertension, ever smoking, hypercholesterolemia). Model 4 adjusts for demographic variables, vascular risk factors, and vascular and inflammatory blood markers (i.e. hsCRP, HDL, IL-6, HOMA-IR, and creatinine).

## Supporting Information

Table S1 **Displays partial correlations (*r*_p_) between Body Mass Index (BMI) and white matter regions, controlling for age.** Bolded r_p_ values reflect significant values with coefficients greater than .2.
(DOCX)
