# Supplementary material for: Body Mass and White Matter Integrity: The Influence of Vascular and Inflammatory Markers
Source: PLoS One. 2013 Oct 16;8(10):e77741. doi: 10.1371/journal.pone.0077741 (PMC3797689; doi:10.1371/journal.pone.0077741)
Supplement: Table S1 — Displays partial correlations (rp) between Body Mass Index (BMI) and white matter regions, controlling for age. Bolded rp values reflect significant values with coefficients greater than .2. (DOCX) [file pone.0077741.s001.docx]

STable1. Partial correlations between body mass and white matter regions.

|  | BMI | |
| --- | --- | --- |
|  | *r*_p_ | *p*-value |
| **Genu of the Corpus Callosum** | **-0.30** | ***p*<.001** |
| **Body of the Corpus Callosum** | **-0.30** | ***p*<.001** |
| **Splenium of the Corpus Callosum** | **-0.31** | ***p*<.001** |
| Anterior Corona Radiata | -0.16 | *p*=.05 |
| Posterior Corona Radiata | -0.03 | *p*=.70 |
| Superior Corona Radiata | 0.00 | *p*=.97 |
| Posterior Thalamic Radiation | -0.13 | *p*=.13 |
| Anterior Internal Capsule | -0.18 | *p*=.03 |
| Posterior Internal Capsule | 0.12 | *p*=.18 |
| External Capsule | -0.17 | *p*=.04 |
| Hippocampal Section of Cingulum | -0.15 | *p*=.08 |
| **Cingulate Section of Cingulum** | **-0.30** | ***p*<.001** |
| Cerebral Peduncles | -0.12 | *p*=.15 |
| Sagittal Stratum | -0.11 | *p*=.19 |
| Superior Fronto-Occipital Tract | -0.09 | *p*=.31 |
| Uncinate Fasciculus | -0.08 | *p*=.30 |
| Superior Longitudinal Fasciculus | -0.04 | *p*=.68 |
| **Fornix Body/Stria Terminalis** | **-0.35** | ***p*<.001** |
